# Chronic Use of Synthetic Cannabinoids Is Associated With Impairment in Working Memory and Mental Flexibility

**DOI:** 10.3389/fpsyt.2020.00602

**Published:** 2020-06-30

**Authors:** Koby Cohen, Yaniv Mama, Paola Rosca, Albert Pinhasov, Aviv Weinstein

**Affiliations:** ^1^Department of Behavioral Science, Ariel University, Ariel, Israel; ^2^Ministry of Health (Israel), Jerusalem, Israel; ^3^Adelson School of Medicine, Ariel University, Ariel, Israel; ^4^Department of Molecular Biology, Ariel University, Ariel, Israel

**Keywords:** synthetic cannabinoids, response inhibition, mental flexibility, emotional processing, cannabis

## Abstract

**Background:**

We have recently shown that chronic use of Synthetic Cannabinoids (SCs) has been associated with mood disorders and impairments in executive functions. There is also evidence indicating that chronic SC users have higher rates of comorbidity with depression and psychotic symptoms. Here, we investigate performance on executive function and emotional processing tasks in regular SC users and a measure of schizotypal traits.

**Method:**

Thirty chronic SC users, 32 recreational cannabis users, and 32 non-using control participants, without history of mental disorder, or current substance abuse diagnosis (mean age 26 ± 4.27 years; 85 males, 9 females), were tested in addiction treatment centers in Israel. Computerized neurocognitive function tests; the N-back task, Go/No-Go task, Wisconsin Sorting Card-like Task (WSCT), and emotional face recognition task and questionnaires of depression, anxiety and schizotypal traits and symptoms were used.

**Results:**

SC users have performed worse than recreational cannabis users and non-cannabis users on the N-back working-memory task (lower accuracy) and the WSCT cognitive flexibility task. SC users showed greater schizotypal traits and symptoms compared with recreational cannabis users and non-user control participants. A positive association was found in cannabinoid-user groups between schizotypal traits and symptoms and cognitive and emotional processing measures. Finally, SC users have scored higher on depression and state-trait anxiety measures than recreational cannabis users or healthy control participants.

**Conclusions:**

Repeated use of SCs is associated with impairment in executive functions and emotional processing. These alterations are associated with depression and schizotypal traits and symptoms. This adds to existing evidence on the long-term consequences of SC drugs and their risks for mental health.

## Introduction

There is a growing use of novel psychoactive substances (NPSs) which contain various psychoactive agents (1, 2). Some of these NPSs contain Synthetic Cannabinoid (SC) compounds which are marketed as a natural herbal mixture under different brands names (3–5). These drugs are composed of multiple types of extremely potent synthetic cannabinoid-agonists as well as additional psychoactive constituents, of which some are unknown (5, 6). The intoxicating effects of SC drugs are similar to the effects of cannabis, commonly with; SC drugs induce reactions such as relaxation, euphoria, perceptual disturbances, and alterations in cognitive abilities (7–9). Importantly, the adverse effects in terms of duration and severity of SCs are more intense than natural cannabis. SC use has been linked with a range of undesired physiological effects ranging in intensity, from nausea to more severe symptoms such as psychomotor agitation, diaphoresis, and palpitations (10, 11). Furthermore, converging evidence has shown an association between SC use and severe affective alterations and cognitive deficits (3, 12). Although SC drugs are gaining popularity, the information regarding their adverse effect and long-term impact on health is limited as well as the general awareness regarding the damaging potential of these drugs (13, 14).

Similar to herbal cannabis, SCs induce their effect through the activation of cannabinoid receptors (CB_1_ and CB_2_) within the Central Nerves System (CNS) (15). In contrast to the psychoactive and non-psychoactive compounds in herbal cannabis such as Δ-9-tetrahydro-cannabinol (THC) and Cannabidiol (CBD) (3–5), SC drugs contain a mixture of psychoactive ingredients, which are more potent and efficacious at the CB_1_ and CB_2_ receptors (16–18). Therefore, although SC drugs are designed to mimic the effect of cannabis, their effects even in low doses are more severe, persistent and unpredictable (8, 19, 20).

There is an agreement that the central psychoactive effect of cannabinoid-based drugs is exerted through direct stimulation of CB_1_ receptors (21, 22). These receptors are observed in high densities in brain regions including the prefrontal cortex, hippocampus, basal ganglia, anterior cingulate, and cerebellum (23). An activation of CB_1_ receptors induces alterations in the release of neurotransmitters and amino-acids in a wide range of neural networks in sub-cortical and cortical brain regions (20). As CB_1_ receptors interact with additional neurotransmitter systems, cannabinoids exert their effects on a variety of cognitive functions, emotional processing, sensory perception and regulation of incoming sensory information (24).

When administered acutely, CB_1_ agonist agents such as THC, the main psychoactive compound of cannabis and different types of SCs, can impair cognitive function as well as emotional processing (20, 25–27). Such effects were observed in animal and human studies (12, 28). Several studies have shown that acute administration of cannabinoid-agonists alters the ability to recognize emotions in others and may induce anxiety symptoms (25, 26, 29). Furthermore, D’Souza and colleagues have reported dose-related adverse effects which were induced following acute consumption of THC in healthy participants; THC has produced a broad range of transient symptoms, including anxiety symptoms and cognitive deficits in healthy individuals that resemble some aspects of psychosis (26). Bedi and colleagues have conducted a well-designed, double-blind, randomized clinical study, and they have reported a dose-related cognitive decline that has been observed in current cannabis users following a treatment with Nabilone (30). More recently, Theunissen and colleagues showed that acute consumption of SC JWH-018 has induced poor coordination, memory deficits, and perception alteration in current cannabis users (20).

The long-term effects of cannabinoid-agonists on cognitive and emotional functions in humans is both mixed and limited, and it is mostly focused on herbal cannabis (27, 31, 32). Neuro-imaging studies have shown that repeated use of cannabis was associated with structural and functional alterations in the CNS. Interestingly, alterations were observed in pre-frontal and limbic regions that are involved in cognitive and emotional processing functions (33). However, while behavioral manifestations of these neuronal alterations in humans are modest (27, 31–33), pre-clinical studies have shown that chronic treatment with cannabinoid-agonists such as SCs has caused severe and persistent cognitive impairment as well as an affective imbalance (34–37). These studies have indicated an association between repeated treatment with cannabinoid-agonists and cognitive deficits in a wide range of domains including; attention, working memory and cognitive flexibility (12). Moreover, treatment with cannabinoid-agonists has induced depression-like and anxiety-like states, and chronic treatment with CB_1_-receptor agonists are considered as applied animal models for affective disorders (38–40). Interestingly, the disruptive effects of cannabinoid-agonists were associated with exposure at an early age, genetic predisposition, and with a higher dosage (34, 41–43).

Clinical reports have indicated a similar phenomenon, in which severe affective disturbances and cognitive deficits were observed among chronic SC users. Cengel and colleagues have shown generalized cognitive impairments in SC users (44). Still, most of the reports regarding this association are based on self-report measurements and surveys (8, 45, 46). Castellanos and Thornton have reported that young SC users have experienced alterations in short-term memory with their main symptom; a severe psychotic episode (47). Additional studies have reported similar clinical symptoms including severe deficits in cognitive functions and psychosis (19, 48, 49). The association between cannabinoids and psychosis is well established (50, 51). There is accumulating evidence of an association between cannabinoids use and psychotic proneness, yet a causal relationship between these two factors is missing (52). Some authors have suggested that psychotic proneness may underline an individual’s genetic predisposition, moderates the adverse effects of cannabis (53, 54). Accordingly, several studies have shown a correlation between cannabinoids use and schizotypal traits which appear to represent individual psychotic proneness among students (55–57). Moreover, schizotypal traits have been associated with greater psychotic experiences and worse undesired effects of cannabis intoxication (58, 59).

In our previous study, we have shown initial evidence for impairments of Working Memory (WM), response inhibition and long-term memory among SC users, compared with non-synthetic cannabis users and healthy control participants (60). SC users displayed lower accuracy and longer reaction time on performance of cognitive tasks compared with non-users and cannabis users (60). In a further study, we have shown a WM impairment in SC users which was associated with structural and functional deficits in several brain regions including the middle frontal gyrus, frontal orbital gyrus, inferior frontal gyrus, insula, anterior cingulate cortex, and the precuneus. Surprisingly, response inhibition in SC users was preserved compared with healthy participants (61). In both studies, SCs have shown depression alongside these cognitive impairments, yet we were unable to control possible confounding factors such as educational levels (60, 61). Altogether, these studies have indicated that SC consumption is associated with severe cognitive impairments, while, there is disagreement regarding the specific cognitive distortion associated with repeated SC use. Moreover, although there is strong evidence for emotional disturbances associated with repeated SC use, there are no studies on emotional processing in SC users. Finally, no study has shown any association between cognitive and emotional function and psychosis proneness in SC users. The purpose of the current study was to expand existing knowledge regarding the effects of SCs on executive function and emotional processing. In addition, we aimed to explore the possible association of these functions with schizotypal traits. We have hypothesized that chronic use of SC would be associated with poorer performance on WM and on response inhibition and cognitive flexibility tasks compared to recreational cannabis users and non-users. Furthermore, we have expected SC users to show worse performance on the emotional processing task compared with performance of both control groups. Finally, we have hypothesized that executive function and emotional processing impairments would be associated with schizotypal proneness among cannabinoid users and not among healthy control participants.

## Methods

### Ethical Approvals

The Institutional Review Board of Ariel University and the Israeli Ministry of Health Office have approved the study. All participants have volunteered to participate in the study and they did not get any incentives for their participation. All the participants have signed an informed consent prior to participation.

### Participants

Ninety-four participants were recruited for the study, including 85 males and 9 females. The mean age was 26.01 (SD = 4.26) years. The total sample was divided into three groups based on their self-reported substance use history: (a) SC users (b) recreational cannabis users and (c) non-users. Both regular cannabis users and non-users were recruited by using convenient snowball sampling *via* friends, relatives or advertisements in social networks. SC users were recruited from three drug addiction treatment inpatient units supervised by the Israeli Ministry of Health located in Ashdod, Eilabun, and Malcishua in Israel. All the participants were administered a screening interview that covered the following areas: medical history, illicit drug use, current psychiatric status, personal psychiatric history and native language. The screening interview, the explanation of the procedure, and the data collection were conducted by a licensed Psychologist (KC).

### Synthetic Cannabinoids-Users

The SC users’ group was initially comprised of 38 participants, 36 males and 2 females, who have frequently consumed SC drugs over the last 2 years. We have defined the inclusion criteria for SC users as regular SC use on a monthly basis, with minimal usage of at least 10 times in the last year and without binge consumption defined as more than 4 usages of SC during the last month. Eight male participants from this group have not completed the experiment and were excluded following the initial screening interview and their data were excluded, thus, the group was finally composed of 30 participants. The mean age of the remaining 30 participants was 26.97 years (SD = 4.17). All participants were evaluated and diagnosed by a senior Psychiatrist prior to the experiment. They were confirmed as not suffering from current psychosis, having co-morbidity with other psychiatric or neurological disorders or a past or current substance use disorder other than cannabinoids.

### Recreational Cannabis-Users

The recreational cannabis users’ group has included 32 participants (28 males and 4 females), who consumed cannabis for recreational purpose more than 10 times in the last year and have never consumed SCs. The mean age in the cannabis user group was 26.99 (SD = 4.17) years. Two participants have not finished the emotional processing task, and the missing data was omitted from related analyses. Exclusion criteria for cannabis participants were history of neurological or psychiatric disorder and history or current substance use disorder.

### Non-Users

The group of non-users has included 29 males and 3 females, altogether 32 healthy individuals, who have reported that they did not consume cannabinoid-based drugs during the past 2 years and have never consumed SCs. Participants’ mean age was 25.41 (SD = 4.53) years. One participant has not finished the emotional processing task, the missing data was omitted from related analyses. Exclusion criteria for healthy control participants were history of neurological or psychiatric disorder and history or current substance use disorder.

### Materials, Stimuli, and Design

#### Demographic and Self-Reported Questionnaires

The demographic questionnaire included items on education level, age, and gender. The questionnaire also contained items regarding the use of psychoactive substances, focusing on cannabinoid-based drugs, and including additional psychoactive substances, tobacco, and alcohol. The date of the last use, frequency of past week, past month and frequency of past year drug use were also assessed. In addition, for measuring psychotic proneness, participants have completed self-reported measures of Schizotypal Personality Questionnaire (SPQ-B) (62, 63). The internal consistency of the SPQ-B ranged from 0.75 to 0.83. In this study, the SPQ-B had a Cronbach internal reliability of α = 0.86. Furthermore, participants have answered the Beck Depression Inventory (BDI) (64) (Cronbach α = 0.86), and the Spielberger state (Cronbach α = 0.86)-trait (Cronbach α = 0.86) anxiety inventory (STAI-S, STAI-T) (65).

#### Executive Function Measures (EF)

For assessing EF, we used computer versions of three tasks which measure; (a) response inhibition (b) WM, and (c) cognitive flexibility (66). (a) The Go/No-Go task was used for assessing response inhibition and sustained attention. In this task participants are required to tap on a corresponding key when “Go” stimuli (blue rectangles) are presented, and to inhibit responses when “No-Go” stimuli (black rectangles) are presented (67, 68). The task has included 150 trials, and the probabilities of occurrence of “Go” and “No-Go” stimuli were equal and randomized (68). The task’s measures RTs, and two types of errors; (1) commission errors (percentages of non-responses for “go” stimuli) (2) omission errors (percentages of responses for “no-go” stimuli). Increased commission or omission error rates in the task have indicated greater impulsivity or sustained attention impairments (69).

(b) The n-back task is considered a “gold-standard” measure for WM function and it consists of alternating conditions with two WM load levels: 1-back and 2-back (70). In the 1-back condition, participants are required to decide if a stimulus on the screen is identical to the previous stimulus. In the 2-back condition, participants are required to decide if a stimulus on the screen is identical to the stimulus presented two steps beck. Accuracy percentages of the two conditions are recorded (71). The two conditions of the n-back represent measures of WM at low and high load (72), in our previous work we have demonstrated WM deficits in SC users, in both 1-back and 2-back conditions (60).

(c) A modified short version of the Wisconsin Card Sorting-like Task (WCST) (73) was used for measuring cognitive flexibility. The short version of the WCST includes 64 response cards and 4 stimulus cards. The stimulus cards are presented in a standard left-to-right order, while response cards are presented one by one according to a specific criterion (color, shape, or number). In the sorting task, the response card should correspond to a feature of the target card. After a sequence of 10 correct responses, the sorting criterion changes and a new sorting criterion must be discovered. The task includes 64 trials. The following indices were recorded; (a) number of completing sets, (b) number of maintaining set failures (c) number of perseveration errors (set-shifting failures), and (d) number of non-perseveration errors. These indices are associated with chronic consumption of cannabis and were observed in schizophrenic patients (74, 75).

#### Emotional Processing

The static facial affect recognition task was used to assess emotion recognition (76). During this task, participants are required to recognize different types of facial expressions of five emotions: happiness, sadness, anger, disgust, fearfulness, and neutral facial expressions of 4 different faces (2 males and 2 females). We have calculated participants’ proportion of accuracy, false alarms, sensitivity (Pr) and response bias (Br) in each emotion (76, 77).

## Results

### Statistical Analysis

The analysis of the results was performed on a Statistical Package for Social Science (SPSS) for windows v.21 (IBM Corp. Armonk, NY, USA). There were three cases of missing data, all missing values were excluded from the analysis. Differences among groups in terms of gender were tested using chi-square test. The group effects on cognitive and emotional processing measures were analyzed with univariate Analysis of Variance (ANOVAs); Bonferroni corrections for t-test were used for *post hoc* group comparisons. In a further analysis, demographic variables and depression, anxiety and tobacco consumption were added as covariates to the ANOVA, in order to investigate the possibility of confounding variables. In order to examine the relationships between age of first cannabinoid use and cognitive performances further Pearson correlations were computed separately for SC and cannabis user groups. Pearson correlations were computed separately for each group in order to explore correlation between SPQ-B, cognitive performance and emotional processing factors.

### Sample Characteristic and Substance Use History

Participants’ drug use history and demographic data are described in **Table 1**. The groups did not significantly differ by gender (χ^2^ = 6.11, *p* > 0.05), age [*F* (2, 91) = 1.46, *p* = 0.32], education level [*F* (2, 91) *=* 1.49, *p* = 0.53] or by alcohol use history [*F* (2, 90) = 1.17, *p* = 0.31]. While there were no differences in tobacco consumption between cannabis and non-users [*t* (61) = 0.69, *p* = 0.77], SC users have consumed more tobacco than non-users [*t* (60) = 12.16, *p* < 0.01] and recreational cannabis users [*t* (59) = 11.49, *p* < 0.01]. SC users have used cannabinoid-based drugs earlier in life than recreational cannabis users [*t* (60) = 2.19, *p* < 0.05]. However, there were no differences between the groups in cannabinoid-consumption frequencies during the last year [*t* (59) = 0.66, *p* = 0.13].

**Table 1:** Demographic and participants characteristics for each group.

	Synthetic	Cannabis	None	Significance
N, frequencies (male: female)	30 (28:2)	32 (28:4)	32 (29:3)	*p* = 0.73
Age, mean (SD)	25.93 (4.27)	27.71 (3.15)	25.4 (4.53)	*p* = 0.32
Education level (sd)	11.96 (1.29)	12.21 (0.69)	12.12 (0.55)	*p* = 0.53
Alcohol consumption (SD)	3.17 (2.72)	4.25 (3.12)	4.15 (3.14)	*p* = 0.31
Tabaco consumption (SD)	19 (8.23)	2.37 (4.75)	1.4 (2.83)	*p* < 0.001
Age of first use for cannabinoids	17.3 (4.61)	19.17 (2.87)	–	*p < 0.05*
Age of first use for SC	22.9 (5.7)	–	–	*–*
Age of first use for cannabis	17.34 (4.1)	19.17 (2.87)	–	*p = 0.05*
Frequency of cannabinoids use during the last year	202.68 (145)	186.87 (135.46)	–	*p* = 0.13
BDI, mean (SD)	40.17 (9.18)	24.93 (5)	25.90 (6.88)	*p <* 0.001
STAI trait, mean (SD)	49.44 (7.98)	34.04 (7.30)	35.21 (9.7)	*p <* 0.001
STAI state, mean (SD)	49.39 (9.75)	31.53 (9.11)	32.03 (10.03)	*p <* 0.001
SPQ-B, mean (SD)	11.66 (4.37)	5.5 (4.38)	4.81 (4.26)	*p < 0.001*

Age and education level reported in years; Alcohol consumption habits drink defined as glass of wine or 250 ml of beer or one shot of alcoholic beverages; Tabaco consumption, cigarettes per day; BDI, Back depression inventory scores; STAI, Silberberg Trait or State anxiety inventory scores; SPQ-B, Schizotypal Personality Questionnaire Brief; significant level of difference between drug groups within the total sample; n.s., non-significant difference.

There was a main effect of group on depression, anxiety and schizotypal trait measurers. SC users had greater scores on the SPQ-B than non-users [*t* (60) = 6.26, *p* < 0.01] and recreational cannabis users [*t* (59) = 5.63, *p* < 0.01], No differences was found in SPQ-B between non-users and recreational cannabis users [*t* (62) = 0.63, *p* = 0.8].

SC users have scored higher on the BDI than non-users [*t* (60) = 7.77; *p* < 0.01] and recreational cannabis users [*t* (59) = 8.31, *p* < 0.01] but there were no differences on BDI score between non-users and recreational cannabis users [*t* (62) = 0.54; *p* =1]. SC users had higher scores on the STAI Trait and State compared with non-users [*t* (60) = 6.89, *p* < 0.01; *t* (60) = 6.6, *p* < 0.01] and recreational cannabis users [*t* (59) =7.01, *p* < 0.01; *p* < 0.01; *t* (59) = 7.15, *p* < 0.01]. There were no differences in STAI State and Trait scores between non-users and recreational cannabis users [*t* (62) = 6.6, *p* = 1; *t* (62) = 0.21, *p* = 0.83].

### Cognitive Performance

#### The Go/No-Go Task

##### Reaction Time

A one-way ANOVA was conducted to explore the effect of group (SC, recreational cannabis users, non-users) on RTs in each condition. Results reveal a main group effect [*F* (2, 90) = 10.95, *p* < 0.001]. SC users were significantly slower in their responses than non-users [*t* (60) = 3.43 *p* < 0.001] and cannabis users [*t* (59) = 4.34, *p* < 0.001; *t* (59) = 3.9, *p* < 0.001]. There were no differences in reaction times of non-cannabis users and recreational cannabis users [*t* (61) = 0.38, *p* = 0.75] (**Table 2**). This effect remained significant when anxiety [*F* (2, 88) = 5.63, *p* < 0.01], depression [*F* (2, 88) = 6.7, *p* < 0.01], and schizotypal trait [*F* (2, 88) = 6.4, *p* < 0.01] were used as covariates. However, this effect was diminished when consumption of cigarettes with tobacco [*F* (2, 88) = 1.1, *p* = 0.33] was entered as a covariate.

**Table 2:** Means (standard deviations) of performance on the Go/No Go task in SC, cannabis users and non-user group.

Go/No Go	Group			Comparison	
	SC	Cannabis	Non-users	*F*(2,90)	*P*-value
Reaction time	508 (147.08)	399.78 (59.52)	406.34 (76.54)	10.95	*p* < 0.001
Omission	1.04 (1.47)	0.64 (1.51)	0.19 (0.64)	3.50	*p* < 0.05
Commission	1.11 (1.84)	0.9 (1.56)	0.75 (1.1)	0.43	*p* < 0.65

Values express mean (SD), reaction time are in milliseconds, errors reported as percentages.

##### Commission and Omission Errors

Analysis has revealed a main group effect on rate of omissions in the Go/No-go task [*F*(2,90) = 3.5, *p* < 0.05]. SC participants have made more omission errors than non-users [*t*(60) = 2.98, *p* < 0.05]. There were no differences in omission errors between recreational cannabis users and SC users [*t*(59) = 1.04, *p* = 0.3] and non-users [*t*(61) = 1.5, *p* = 0.13]. This effect remained significant when anxiety [*F*(2,88) = 3.77, *p* < 0.05], depression [*F*(2,88) = 1.42, *p* < 0.05] and schizotypal trait [*F*(2,88) = 3.16, *p* < 0.05] were used as covariates, yet, it was no longer significant when consumption of cigarettes with tobacco [*F*(2,88) = 1.41, *p* = 0.24] was used as a covariate. Further analysis has failed to show differences between the groups in the rate of commission errors [*F*(2,90) = 0.43, *p* = 0.64] (**Table 2**).

#### The N-Back Task

For the analysis of WM performances, accuracy data were analyzed using a repeated measures ANOVA with group (SC, recreational cannabis, and non-users) as the between-subject factor and memory load (1-back, 2-back) as the within-subject factors. Results have revealed a significant main effect for memory load, [*F* (1, 90) = 82.75, *p* < 0.001]. The accuracy scores in the 1-back condition were significantly higher than the accuracy scores of the 2-back condition [*t* (92) = 8.91, *p* < 0.001]. Additionally, a main group effect was observed, [*F* (2, 90) = 18.52; *p* < 0.001]. Post hoc analyses with Bonferroni corrections has revealed that SC users were significantly less accurate than both non-users [*t* (60) = 5.67, *p* < 0.001] and recreational cannabis users [*t* (61) = 4.80, *p* < 0.01]. There was no difference in accuracy between recreational cannabis users and non-users [*t* (62) = 0.83, *p* = 1] (**Table 3**). The effect on accuracy remained significant when tobacco [*F* (2,88) = 5.53, *p* < 0.01], schizotypal trait [*F* (2,88) = 5.58, *p* < 0.01], anxiety [*F* (2,88) = 7.23, *p* < 0.01], and depression [*F* (2,88) = 3.7, *p* < 0.05] were used as covariates.

**Table 3:** Accuracy of N-back performance by group.

	Group			Comparison	
	SC	Cannabis	None	*F*(2,90)	*P*-value
1-back	86.09	91.15	92.39	18.52	*p* < 0.001
	(7.4)	(4)	(2.68)		
2-back	79.05	87.211	87.87		
	(9.25)	(6.09)	(4.8)		

Accuracy values are expressed as mean correct percentage (SD).

#### The Wisconsin Sorting Card-Task

##### Analysis of Number of Completing Sets

There was a main group effect on the number of completing sets [*F* (2, 91) = 35.84, *p* < 0.001], SC had completed less sets (M = 1.66, SD = 1) than non-users (M = 3.96, SD = 4.03) [*t* (60) = 8.17, *p* < 0.001] and recreational cannabis users (M = 3.75, SD = 1.29) [*t* (60) = 6.82, *p* < 0.001]. No differences in number of completing sets between non-users and recreational cannabis users [*t* (62) = 0.72, *p* = 1]. The main effect remained significant in further ANCOVAs when tobacco-cigarette consumption [*F* (2, 89) = 14.24, *p* < 0.01], schizotypal trait [*F* (2, 88) = 14.75, *p* < 0.01], anxiety [*F* (2, 88) = 16.15, *p* < 0.001], and depression [*F* (2, 89) = 10.7, *p* < 0.01] were used as covariates.

##### Analysis of Maintaining Set Failures

There was a main group effect on maintaining set failures [*F* (2, 91) = 3.43, *p* < 0.05], SC had performed more failures in maintaining sets (M = 1, SD = 1.20) than non-users (M = 0.34, SD = 0.75) [*t* (60) = 2.6, *p* < 0.05]. There was no difference between SC users and recreational cannabis user (M = 0.56, SD = 0.75) [*t* (60) = 1.72, *p* = 0.19] and non-users vs. recreational cannabis users [*t* (62) = 1.63, *p* = 1]. This main effect remained significant in further ANCOVAs when tobacco cigarette consumption [*F* (2, 89) = 14.24, *p* < 0.01] and anxiety [*F* (2, 88) = 16.15, *p* < 0.001], were used as covariates. However, this effect was reduced to a trend when depression was used as a covariate [*F* (2, 89) = 2.5, *p* = 0.08] and it was diminished when schizotypal scores were entered as a covariate [*F* (2, 88) = 2.1, *p* = 0.12].

##### Analysis of Non-Perseverative and Perseverative Errors

A one-way ANOVA has indicated a main group effect on non-perseverative errors [*F* (2, 91) = 43.58, *p* < 0.01] and perseverative errors [*F* (2, 91) = 19.98, *p* < 0.01]. SC users had performed more non-perseverative errors (M = 12.86, SD = 5.07) and perseverative errors (M = 11.53, SD = 3.76) than non-users (M = 5.65, SD = 2.75; M = 6.09, SD = 2.58) [*t* (60) = 8.16, *p* < 0.001; *t*(60) = 5.83, p < 0.001] and recreational cannabis users (M = 5.71, SD = 2.6; M = 7, SD = 4.28) [*t* (59) = 8.80, *p* < 0.01; *t*(59) = 4.76, *p* < 0.01]. There were no differences in these measurers between non-users and recreational cannabis users [*t* (61) = 0.07, *p* = 1; *t* (61) = 1.09, *p* = 1]. The group effect on error rates remained significant in further ANCOVAs when consumption of cigarettes with tobacco [*F* (2, 89) = 13.24, *p* < 0.001], schizotypal trait [*F* (2, 88) = 19.56, *p* < 0.001] anxiety [*F* (2, 88) = 23.05, *p* < 0.001] and depression [*F* (2, 89) = 13.5, *p* < 0.01], were used as covariates.

#### Emotional Processing Task

A repeated measures ANOVA was conducted on hit rates, false alarms, Pr, and Br with six emotions (anger, sadness, disgust, happiness, fear, and neutrality) as within-subject factors and group (non-users, cannabis users, SC users) as the between-subject factor.

### Hit Rates

**Figure 1:**
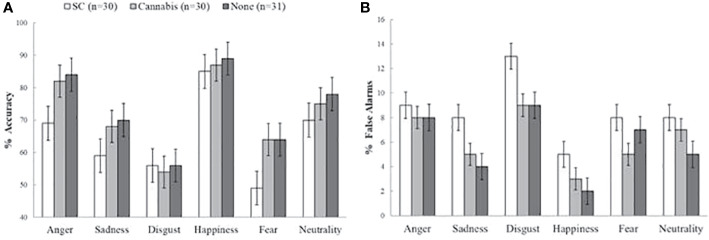
Accuracy and false alarm rates on the emotion recognition task. **(A)** Main effect of group on accuracy (mean percentage of correct responses). Synthetic cannabinoids (SC) users were less accurate compared to non-users and marginally less accurate than natural cannabis users. **(B)** Main group effect on false alarms (mean percentage of false alarms). SC users had more false alarms than non-users, and marginally more false alarms than natural cannabis users. The error bars represent standard error of the mean.

There was a main group effect on hit rates [*F* (2, 88) = 4.6, *p* < 0.05]. Post-hoc tests has indicated that while there was no difference between non-users (M = 0.73, SD = 0.07) and recreational cannabis users (M = 0.71, SD = 0.12) [*t* (61) = 0.6, *p* = 1], SC users had less hits (M = 0.62, SD = 0.14) compared with non-users [*t* (58) = 4.23, *p* < 0.01] and marginally less than accurate then recreational cannabis users [*t* (59) = 4.98, *p* = 0.08]. There was not interaction between group and emotion [*F* (10, 440) = 0.38, *p* = 0.25]. The group effect on hit rates remained significant in further ANCOVAs when consumption of cigarettes with tobacco [*F* (2, 86) = 4.61, *p* < 0.05] and anxiety [*F* (2, 85) = 3.83, *p* < 0.05] were used as covariates. This was no longer significant when depression [*F* (2, 86) = 1.03, *p* = 0.35] or schizotypal score were used as covariates [*F* (2, 88) = 1.12, *p* = 0.32] (**Figure 1A**).

### False Alarms

There was a main group effect on false alarms [*F* (2, 88) = 338.74, *p* < 0.001] (**Figure 1B**). Post-hoc tests has indicated that SC-users had more false alarms (M = 0.09, SD = 0.06) than non-users (M = 0.06, SD = 0.016) [*t* (58) = 2.74, *p* < 0.05] and marginally more false alarms then recreational cannabis users (M = 0.06, SD = 0.02) [*t* (59) = 2.3, *p* = 0.07]. There was no difference in false alarms between recreational cannabis users and non-users [*t* (61) = 0.7, *p* = 1]. Finally, there was not interaction effect of group and emotion [*F* (10, 440) = 0.65, *p* = 0.72]. The group effect on false alarms rates remained significant in further ANCOVAs when consumption of cigarettes with tobacco [*F* (2, 86) = 5.74, *p* < 0.01], and anxiety [*F* (2, 85) = 3.21, *p* < 0.05] were used as covariates. This was no longer significant when depression [*F* (2, 86) = 1.13, *p* = 0.36] or schizotypal [*F* (2, 88) = 1.12, *p* = 0.33] were used as covariates.

### Sensitivity

**Figure 2:**
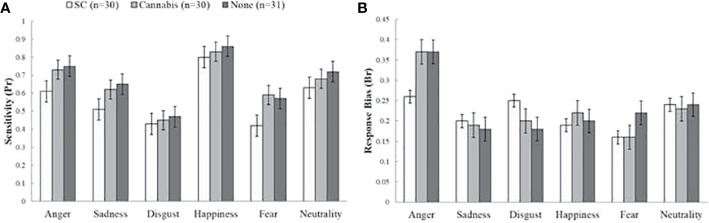
Sensitivity (Pr) and response bias (Br) values on the emotional processing task. **(A)** Main effect of group on sensitivity (Pr). Synthetic cannabinoids (SC) users showed less sensitivity than non-users and marginally lower score than natural cannabis users. **(B)** No differences between groups in response bias index (Br). The bars represent mean scores ± standard error of the mean.

There was a main group effect on Pr rates [*F* (2, 88) = 4.67, *p* < 0.05] (**Figure 2A**). Post-hoc tests indicated that SC-users showed less sensitivity (M = 0.55, SD = 0.18) than non-users (M = 0.66, SD = 0.09) [*t* (58) = 2.88, *p* < 0.05] and marginally lower scores then recreational cannabis users (M = 0.64, SD = 0.14) [*t* (59) = 2.27, *p* = 0.07]. There were no differences between recreational cannabis users and non-users [*t* (61) = 0.6, *p* = 1]. Finally, there was no interaction effect of group and emotion [*F* (10, 440) = 1.42, *p* = 0.33]. The effect of group on Pr rates remained significant in further ANCOVAs when tobacco [*F* (2, 86) = 5.03, *p* < 0.001], and anxiety [*F* (2, 85) = 4.23, p < 0.01] were used as covariate factors. Yet, the effect was diminished when depression [*F* (2, 89) = 1.17, *p* = 0.31] or schizotypal [*F* (2, 88) = 1.87, *p*=0.31] were used as covariates.

### Response Bias

There was a main effect of emotion type on Br score [*F* (5, 440) = 11.34, *p* < 0.01] but analysis of group effects on response bias has shown no significant differences between groups [*F* (10, 440) = 2, *p* = 0.17] (**Figure 2B**).

### Analyses Between Age of First Cannabinoid Use and Tasks Performance

We have investigated the association between age of first cannabinoids use and task performance using simple Pearson’s correlation for SC and cannabis groups. For SC users, there were no significant correlations between age of first cannabis and SC consumption with RTs (*r* = 0.78, *p* = 0.68; *r* = 0.02, *p* = 0.89),omission errors (*r* = −0.09, *p* = 0.622; *r* = 0.03, *p* = 0.85) on the Go-No/Go task, WM performance on the N-back task (*r* = 0.16, *p* = 0.39; *r* = −0.23, *p* = 0.21), number of completing sets (*r* = 0.24, *p* = 0.19; *r* = 0.11, *p* = 0.53), maintaining sets failures (*r* = −0.22, *p* = 0.23; *r* = −0.12, *p* = 0.52) and error rates (*r* = −0.09, *p* = 0.60; *r* = 0.19, *p* = 0.31) on the WCST, and accuracy (*r* = 0.20, *p* = 0.27; *r* = −0.43, *p* = 0.82), false-alarm (*r* = −0.11, *p* = 0.53; *r* = −0.03, *p* = 0.84), sensitivity (*r* = 0.19, *p* = 0.30; *r* = −0.04, *p* = 0.81) or response bias (*r* = −0.17, *p* = 0.35; *r* = −0.06, *p* = 0.72) on the emotional processing task. Similarly, for cannabis users there was no significant correlation between age of first cannabis use and WM performance (*r* = 0.11, *p* = 0.54), RTs (*r* = −0.25, *p* = 0.17) and omission errors (*r* = −0.15, *p* = 0.41) on the Go-No/Go task, number of completing sets (*r* = −0.04, *p* = 0.80), maintaining set failures (*r* = 0.05, *p* = 0.75) and error rates (*r* = 0.04, *p* = 0.81) on the WCST, and accuracy (*r* = −0.31, *p* = 0.09), false-alarm (*r* = 0.22, *p* = 0.23), sensitivity (*r* = −0.31, *p* = 0.09) or response bias (*r* = −0.08, *p* = 0.66) on the emotional processing task.

### Exploratory Analyses Between Schizotypal and Tasks Performances

We have investigated the association between SPQ-B scores and task performance using simple Pearson’s correlation within each group separately. For SC users, there was a negative correlation between schizotypal traits and WM performance (*r* = −0.45, *p* < 0.01). A similar pattern was observed for recreational cannabis users; greater scores on the schizotypal trait scale were positively associated with less accuracy on the WM task (*r* = −0.36, *p* < 0.05). Moreover, for SC users, greater scores on the schizotypal trait scale were associated with poorer accuracy (*r* = 0.45, *p* < 0.05) and less sensitivity (*r* = 0.44, *p* < 0.01) on the emotional processing task. Among non-users there were no significant associations between SPQ-B scores and WM performance (*r* = −0.05, *p* = 0.37), emotional processing accuracy (*r* = −0.13, *p* = 0.23) or sensitivity (*r* = −0.11, *p* = 0.28) measurers.

## Discussion

The current study has shown impairments in mental flexibility of SC users. These cognitive deficits cannot be explained by demographic variables such as age, gender, alcohol consumption or educational levels. Previous studies have indicated a generalized impairment in high-order cognitive function of SC users, impairments which were accompanied with neuronal alterations and depression.

The main findings of this study indicate executive function deficits of chronic SC users. These impairments demonstrate poor accuracy on the n-back task, indicating an impairment of WM. Performance on the WCST task has also shown an impairment of mental flexibility indicated by more errors, less completed categories and more failures to maintain sets. These deficits were not observed in recreational cannabis users or healthy control participants. These results are consistent with our previous findings on WM impairment (60, 61) and with additional human and pre-clinical studies examining the effects cannabinoid-agonists on cognitive function. Cengel and colleagues reported impairments in several cognitive functions such as attention, memory, executive, and visual-spatial functions of SC users that were more severe than individuals with cannabis use disorder and healthy control group (44). Furthermore, SC users made more omission errors on the Go/No-go task, indicating impairment in response inhibition. Further analysis of covariance has indicated that adding depression, anxiety, schizotypal trait, and tobacco consumption as covariates has reduced this effect.

In contrast, negative results were reported by Altintas and colleagues who have examined several cognitive domains in SC users who have experienced psychotic episodes and compared their performance with hospitalized schizophrenic patients. Interestingly, there were no differences between the schizophrenic patients and SC users in cognitive function (78).

Recently, Livny and colleagues have reported WM impairment in SC users that were tested on the n-back test and these impairments were associated with structural and functional deficits in several brain regions including the middle frontal gyrus, frontal orbital gyrus, inferior frontal gyrus, insula, anterior cingulate cortex and the precuneus (61). Yet, the response inhibition ability in SC using the same task as ours was preserved compared with control participants. Our results, together with Livny and colleagues suggest that unlike WM impairments, there is no strong evidence for response inhibition impairment in SC users as this variable was confounded by other variables such as tobacco smoking and depression.

The Pharmacological approach may provide an appropriate explanation for the association between the consumption of cannabinoid-agonists and impairment of cognitive functions (35, 37). Accordingly, a consumption of exogenous CB_1_ receptor agonists may alter CB_1_ modulation of additional neurotransmitters such as dopamine, serotonin and noradrenaline (20, 22, 35). In a pre-clinical study, chronic consumption of THC has led to dopamine receptors down-regulation as well as WM deficits (79). Additional rodent studies have indicated that administration of CB_1_ receptor agonists has induced a decrease in prefrontal serotonin levels in a way which alters cognitive function in general and learning abilities specific (80). Finally, activation of CB_1_ receptors produces an inhibitory effect on GABAergic neurons, an effect which alters the neuronal activity of prefrontal brain regions (81). Furthermore, studies show the inhibitory effects of cannabinoid-agonists on GABA activity in the rat’s frontal cortex, amygdala, hippocampus and cerebellum (82, 83). This inhibition has produced a down-regulation in GABAergic transmission in the prefrontal cortex that is associated with cognitive impairments (83). Altogether, these may explain the wide range of cognitive dysfunction which was observed among SC users.

Impaired emotional processing was observed among SC users compared with regular cannabis users and non-users. The impairment was demonstrated by lower accuracy, more false alarms and lower sensitivity. However, when depression ratings were added as a covariate the effect was diminished. This finding implies that depression has a strong effect on emotional processing and it can explain why SC users have made errors in recognizing facial emotional expressions in other people. It is well established that depressed patients have difficulties in processing facial emotional expressions (84) and we now demonstrate the association between emotional processing and depression in SC users. Our results support previous human and pre-clinical studies which have shown the adverse effect of long-term SC and cannabis consumption on affective states and emotional function (8, 29, 45, 46, 85). On the other hand, in contrast to recent studies (76, 77), in the present study no differences between recreational cannabis users and non-users in emotional processing were found. Several explanations are proposed for this inconsistency. First, this lack of effect may be due to inherent differences in the task designs, for example in contrast to Hindocha and colleagues we did not use emotional faces in different intensity (76), nor dynamic emotion expression faces as Platt and colleagues (77). Second, in the current sample we were able to control for alcohol consumption. This is important since long-term use of alcohol affects emotional processing abilities among cannabis users in previous studies (76).

Finally, we have found a negative correlation between schizotypal traits and WM performance in SC and recreational cannabis user groups. Moreover, for SC users greater schizotypal traits were associated with poorer performance on the emotional processing task, and may have confounded the effect of SC on emotional processing. These associations stand in line with current research which showed that the adverse effects of cannabinoids are partially associated with psychotic proneness (68, 77). The present data may support the last notion and supports the evidence of the involvement of endo-cannabinoid system in the psychopathology of schizophrenia, yet, the current study could not examine the moderation effect of psychosis proneness on the association between SC use and emotional processing.

### Limitations of the Current Study

While interpreting the results of the current study, some potential limitations should be taken into account. First, objective measures of participants’ cannabinoids use as well as other psychoactive compounds were not taken. These assessments may be important since there is a relationship between blood concentrations of those psychoactive ingredients and cognitive function as well as emotional processing (20, 26). However, it is important to take into account that some of these SC drugs are composed of psychoactive ingredients that may not be detected by urine or blood test (17). Furthermore, SC users have consumed regular cannabis as well and the current study could not assess whether the cognitive and emotional deficits presented by SC users are induced due to excessive use of SC rather than the interaction of SC chronic use combined with regular cannabis. Moreover, the current research could not assess whether the observed effects are dose-related or perhaps an expression of a genetic predisposition. In addition, we have reported an elevation of depressive and anxiety symptoms as well as schizotypal traits in SC users. Anxiety and depression have been previously associated with schizotypal symptoms, as well as chronic drug use. SC users were administered a screening interview and psychiatric evaluation in order to exclude participants with a history of neurological or psychiatric disorder. However, the current study could not assess whether the elevation of schizotypal measure in SC users is due to the influence of anxiety or depression or a result of prolonged drug abuse. Prospective studies with more objective measurements and details regarding patients’ substance use and clinical presentations are therefore needed to address these limitations. Furthermore, we have found that depression and consumption of cigarettes with nicotine has reduced the observed effect of SC on response inhibition and emotional processing. These findings indicate that they are confounding variables affecting the association between the use of SCs on cognitive and emotional processing. Finally, the sample size of the current study was relatively small since chronic SC users are a very unique and rare cohort and difficult to recruit. Due to the relatively small sample size we were unable to conduct additional analyses of further potential confounding variables or to infer causality. Future studies may consider using larger samples in order to investigate cognitive, emotional and psychotic proneness among SC users.

## Conclusions

The current study provides further evidence of impaired cognitive and emotional function in chronic SC users. SC users have presented deficits in; WM, mental flexibility and response inhibition. In addition, elevation of depressive and anxiety symptoms as well as schizotypal traits were observed. Some of those cognitive and emotional processing dysfunctions were associated with schizotypal traits in the cannabinoid users’ groups. It is plausible that these deficits are a result of the toxic effects of extremely potent cannabinoids may have on the human’s brain. Yet, further studies are needed to replicate and expand the last conclusions.

## Data AvailabilityStatement

The datasets generated for this study are available on request to the corresponding author.

## Ethics Statement

The studies involving human participants were reviewed and approved by Ministry of Health Jerusalem Israel. The patients/participants provided their written informed consent to participate in this study.

## Author Contributions

All individuals included as authors of the paper have contributed substantially to the scientific process leading up to the writing of the paper. The authors have contributed to the conception and design of the project, performance of the experiments, analysis and interpretation of the results and preparing the manuscript for publication.

## Conflict of Interest

The authors declare that the research was conducted in the absence of any commercial or financial relationships that could be construed as a potential conflict of interest.
